# A Case of Large Bilateral Pulmonary Embolism Presenting Only With Dyspnea in a Primary Care Clinic

**DOI:** 10.7759/cureus.115654

**Published:** 2026-09-02

**Authors:** Abraham Alhatou, Zainab Farooq, Fizza Ahmed, Randy Scott

**Affiliations:** 1 DeBusk College of Osteopathic Medicine, Lincoln Memorial University, Knoxville, USA; 2 Osteopathic Medicine, Lake Erie College of Osteopathic Medicine, Bradenton, USA

**Keywords:** acute hypoxemia, cardiac bnp, dyspnea, pulmonary embolism, shortness of breath, sinus tachycardia, sob - shortness of breath, troponin level

## Abstract

Pulmonary embolism (PE) is a potentially fatal condition with a wide spectrum of clinical presentations. While dyspnea is the most common presenting symptom, the absence of classic features such as chest pain, hypoxia, or syncope may significantly reduce clinical suspicion, particularly in outpatient settings. We report a case of a 77-year-old female who presented to a family medicine clinic with isolated shortness of breath and minimal objective abnormalities, yet was subsequently found to have extensive bilateral central pulmonary emboli with evidence of right ventricular strain. This case highlights the diagnostic challenge of PE presenting with isolated dyspnea and minimal associated symptoms, particularly in a patient with asthma, where a pre-existing pulmonary diagnosis may contribute to anchoring bias and delay consideration of an alternative etiology.

## Introduction

Pulmonary embolism (PE) remains a leading cause of cardiovascular morbidity and mortality, with clinical presentations ranging from catastrophic hemodynamic collapse to subtle, nonspecific symptoms [1]. Shortness of breath is the most common presenting complaint; however, the absence of accompanying features such as chest pain, hypoxia, syncope, or hemoptysis can substantially lower clinical suspicion, particularly in outpatient and primary care settings [2]. As a result, PE may be underrecognized in patients who appear clinically stable at initial evaluation.

Risk stratification tools and symptom-based assessments are frequently used to guide diagnostic decision-making, yet these methods may fail to identify patients with clinically significant thromboembolic disease who lack classic features [3]. This limitation is especially relevant in elderly patients and those with chronic pulmonary conditions, such as asthma, where dyspnea may be attributed to more benign etiologies. Furthermore, normal oxygen saturation or arterial blood gas findings do not reliably exclude PE, as significant clot burden and right ventricular strain may be present despite preserved oxygenation [4].

We present a case of an elderly female who initially presented to a family medicine clinic with isolated dyspnea and minimal objective abnormalities, yet was subsequently found to have extensive bilateral central pulmonary emboli with evidence of right ventricular strain. This case highlights the diagnostic challenges posed by subtle presentations of PE, particularly when underlying asthma provides a plausible alternative explanation for dyspnea, and underscores the importance of maintaining clinical suspicion despite reassuring initial findings.

## Case presentation

A 77-year-old White female with a past medical history significant for asthma and a family history of myocardial infarction presented to a family medicine clinic with acute-onset shortness of breath. She denied chest pain, pleuritic pain, palpitations, syncope, hemoptysis, cough, fever, lower extremity swelling, or recent immobilization. Review of systems was otherwise negative.

At the time of clinic evaluation, vital signs demonstrated a heart rate of 109 beats per minute, blood pressure of 122/81 mmHg, respiratory rate of 20 breaths per minute, and oxygen saturation of 96% on room air. She was afebrile and appeared comfortable at rest. Physical examination revealed clear lung fields without wheezing and no evidence of lower-extremity edema or tenderness. Given her advanced age, persistent dyspnea, and mild tachycardia despite preserved oxygenation and absence of other classic symptoms, she was referred to the emergency department for further evaluation.

In the emergency department, computed tomography pulmonary angiography demonstrated extensive bilateral central pulmonary emboli with findings suggestive of right heart strain (Figure 1). Additional findings included emphysema, patchy bibasilar atelectasis, atherosclerosis, a moderate-sized hiatal hernia, colonic diverticulosis, and multiple pulmonary nodules, the largest measuring 2 mm.

**Figure 1:**
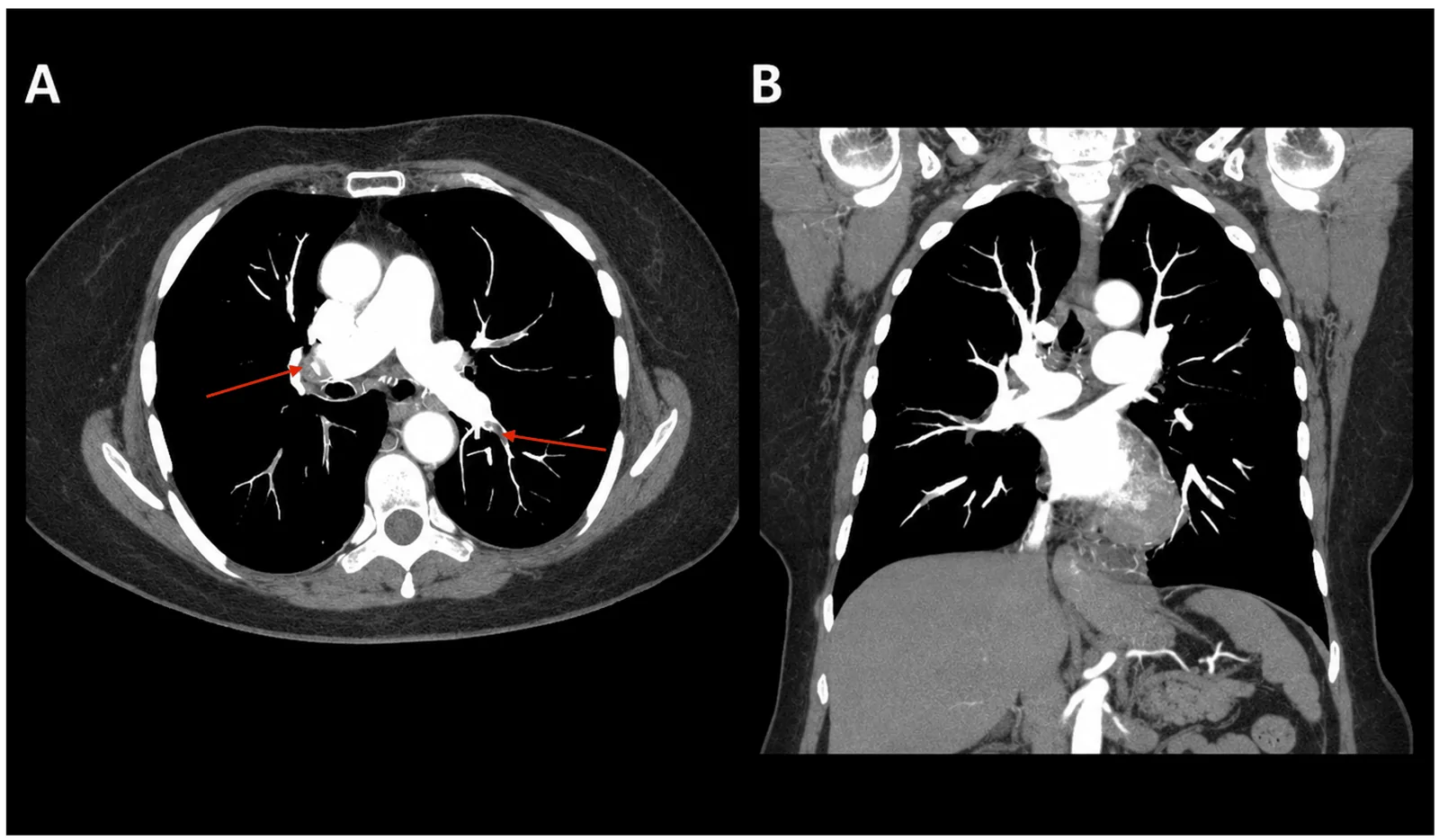
CT pulmonary angiography demonstrates extensive bilateral central pulmonary emboli. Axial (A) and coronal (B) maximum-intensity projection images demonstrate extensive bilateral intraluminal filling defects involving the central pulmonary arteries. Red arrows in panel A indicate representative right- and left-sided pulmonary arterial filling defects.

Laboratory evaluation demonstrated elevated cardiac biomarkers and leukocytosis. The laboratory findings are summarized in Table 1. Bilateral lower-extremity venous duplex ultrasonography revealed an occlusive deep venous thrombosis of the left posterior tibial vein.

**Table 1:** Laboratory findings on presentation.

Laboratory parameter	Patient value	Reference range	Unit
White blood cell count	12.8	4.5-11.0	×10⁹/L
High-sensitivity troponin, initial	165	≤10	ng/L
High-sensitivity troponin, repeat	189	≤10	ng/L
B-type natriuretic peptide (BNP)	138	<100	pg/mL

Transthoracic echocardiography performed two days later demonstrated a preserved left ventricular ejection fraction of 55-60%, with mild right ventricular enlargement and an estimated right ventricular systolic pressure of 41.4 mmHg, consistent with right ventricular strain. The patient remained hemodynamically stable throughout hospitalization and was discharged later that day with appropriate anticoagulation therapy and outpatient follow-up.

## Discussion

This case illustrates a diagnostically challenging presentation of acute PE characterized by isolated dyspnea, preserved oxygen saturation, and minimal initial abnormalities on outpatient evaluation. Despite the absence of classic PE symptoms, the patient was ultimately found to have extensive bilateral central pulmonary emboli with biochemical and imaging evidence of right ventricular strain, meeting criteria for intermediate-risk PE [1,5].

Dyspnea is the most frequently reported symptom of PE; however, it is highly nonspecific and commonly encountered in outpatient practice [2]. In primary care settings, dyspnea is often attributed to chronic pulmonary disease, infection, or cardiac conditions, and anchoring bias may delay recognition of thromboembolic disease [3]. In this case, a history of asthma likely contributed to reduced initial clinical suspicion despite concerning features such as advanced age and tachycardia.

Notably, the patient’s oxygen saturation remained within normal limits, a finding that can falsely reassure clinicians. Prior studies have demonstrated that arterial blood gas abnormalities and oxygen saturation measurements lack sufficient sensitivity to exclude PE, even in patients with significant clot burden [4]. As illustrated in this case, mild tachycardia may represent the only early physiologic abnormality and should prompt careful consideration of PE, particularly in elderly patients.

The presence of elevated cardiac biomarkers and imaging evidence of right ventricular strain classified this patient as having intermediate-risk PE, a category associated with increased morbidity and mortality compared with low-risk disease [1,5,6]. Recognition of this risk profile is essential, as it carries important prognostic and management implications despite an initially benign clinical appearance.

This case underscores the need for outpatient clinicians to maintain a broad differential diagnosis when evaluating unexplained dyspnea, even in the absence of classic PE features. Early referral for further evaluation may be lifesaving, particularly when subtle but concerning clinical findings are present.

## Conclusions

PE may present with isolated dyspnea and minimal objective abnormalities, even in cases of extensive clot burden and right ventricular strain. Normal oxygen saturation does not exclude clinically significant PE. This case highlights the importance of maintaining clinical suspicion for PE in elderly patients presenting with unexplained dyspnea in outpatient settings.

## References

[1] Konstantinides SV, Meyer G, Becattini C (2020). 2019 ESC guidelines for the diagnosis and management of acute pulmonary embolism developed in collaboration with the European Respiratory Society (ERS): the Task Force for the diagnosis and management of acute pulmonary embolism of the European Society of Cardiology (ESC). Eur Heart J.

[2] Goldhaber SZ (2004). Pulmonary embolism. Lancet.

[3] Miniati M, Prediletto R, Formichi B (1999). Accuracy of clinical assessment in the diagnosis of pulmonary embolism. Am J Respir Crit Care Med.

[4] Stein PD, Goldhaber SZ, Henry JW, Miller AC (1996). Arterial blood gas analysis in the assessment of suspected acute pulmonary embolism. Chest.

[5] Becattini C, Agnelli G (2016). Risk stratification and management of acute pulmonary embolism. Hematology Am Soc Hematol Educ Program.

[6] Lankeit M, Konstantinides S (2012). Is it time for home treatment of pulmonary embolism?. Eur Respir J.

